# Deficiency and dysfunctional roles of natural killer T cells in patients with ARDS

**DOI:** 10.3389/fimmu.2024.1433028

**Published:** 2024-08-30

**Authors:** Ki-Jeong Park, Tae-Ok Kim, Young-Nan Cho, Hye-Mi Jin, Young-Goun Jo, Hong-Joon Shin, Bo Gun Kho, Seung-Jung Kee, Yong-Wook Park

**Affiliations:** ^1^ Department of Rheumatology, Chonnam National University Medical School and Hospital, Gwangju, Republic of Korea; ^2^ Department of Pulmonology, Chonnam National University Medical School and Hospital, Gwangju, Republic of Korea; ^3^ Department of Surgery, Chonnam National University Medical School and Hospital, Gwangju, Republic of Korea; ^4^ Department of Laboratory Medicine, Chonnam National University Medical School and Hospital, Gwangju, Republic of Korea

**Keywords:** acute respiratory distress syndrome, natural killer t-cells, bronchoalveolar lavage fluid, macrophages, fibroblasts

## Abstract

**Objective:**

Acute respiratory distress syndrome (ARDS) presents a global health challenge, characterized by significant morbidity and mortality. However, the role of natural killer T (NKT) cells in human ARDS remains poorly understood. Therefore, this study explored the numerical and functional status of NKT cells in patients with ARDS, examining their clinical relevance and interactions with macrophages and fibroblasts during various stages of the syndrome.

**Methods:**

Peripheral blood from 40 ARDS patients and 30 healthy controls was analyzed, with paired samples of peripheral blood and bronchoalveolar lavage fluid (BALF) from seven ARDS patients. We measured levels of NKT cells, cytokines, CD69, programmed death-1 (PD-1), and annexin-V using flow cytometry, and extracellular matrix (ECM) protein expression using real-time PCR.

**Results:**

ARDS patients exhibited decreased circulating NKT cells with elevated CD69 expression and enhanced IL-17 production. The reduction in NKT cells correlated with PaO_2_/FiO_2_ ratio, albumin, and C-reactive protein levels. Proliferative responses to α-galactosylceramide (α-GalCer) were impaired, and co-culturing NKT cells with monocytes or T cells from ARDS patients resulted in a reduced α-GalCer response. Increased and activated NKT cells in BALF induced proinflammatory cytokine release by macrophages and ECM protein expression in fibroblasts.

**Conclusion:**

ARDS is associated with a numerical deficiency but functional activation of circulating NKT cells, showing impaired responses to α-GalCer and altered interactions with immune cells. The increase in NKT cells within BALF suggests their role in inducing inflammation and remodeling/fibrosis, highlighting the potential of targeting NKT cells as a therapeutic approach for ARDS.

## Introduction

Acute respiratory distress syndrome (ARDS), characterized by bilateral pulmonary infiltrates and acute hypoxemia, remains a significant global cause of morbidity and mortality (1). The disease encompasses a series of exudative, proliferative, and fibrotic phases influenced by diverse immune cells (2, 3). Specifically, macrophages and fibroblasts play critical roles throughout these phases (4, 5). In the exudative phase, M1-polarized macrophages drive proinflammatory responses, while myofibroblasts are involved in transferring fibrinous exudate into the alveoli and producing fibronectin (5–7). The subsequent proliferative phase involves M2 macrophages and the synthesis of the extracellular matrix (ECM) orchestrated by myofibroblasts, with imbalances potentially leading to the fibrotic phase (8, 9). Notably, recent studies have highlighted intricate interactions among macrophages, fibroblasts, and natural killer T (NKT) cells in inflammation and fibrosis across various diseases (9–11).

NKT cells are innate-like T cells with characteristics of both conventional T cells and natural killer (NK) cells. They express the invariant Vα24-Jα18 T cell receptor (TCR) chain alongside the Vβ11 TCR chain (12). Upon recognizing glycolipid antigens presented by the major histocompatibility complex (MHC) class I-like molecule CD1d, NKT cells rapidly release a range of cytokines involved in T helper responses, thereby inducing subsequent immune reactions (13). Deficiency and dysfunction in NKT cell cytokine production can impair immune regulation and increase susceptibility to autoimmune diseases, cancer, and infections. Conversely, NKT cells that are numerically and functionally normal can amplify pathogenic immune responses and contribute to the development of diseases like atherosclerosis, graft-versus-host disease, and allergy (12, 14).

The role of NKT cells in lung diseases has been the focus of several studies (15). Experimental murine models of ARDS have demonstrated that activating NKT cells with α-galactosylceramide (α-GalCer) increases proinflammatory cytokine production, exacerbating respiratory failure (16, 17). However, in models of bleomycin-induced pulmonary fibrosis, NKT cells exhibit a protective role by inhibiting the progression of fibrosis (15, 18, 19). These findings suggest that the roles of NKT cells are context-dependent, influenced by factors such as the subjects and tissues studied. Consequently, research on NKT cells in human ARDS is limited, and their specific role in this condition remains poorly understood. Thus, this study aimed to investigate the levels and function of NKT cells in patients with ARDS and their roles in the disease’s pathogenesis.

## Materials and methods

### Patient characteristics

The study comprised 40 ARDS patients (30 males and 10 females, mean age ± SD: 62.8 ± 18.6 years) and 30 healthy controls (HCs, 14 males and 16 females, mean age ± SD: 67.2 ± 4.65 years). The patient cohort was collected between March 2018 and December 2021; however, the patients diagnosed with COVID-19 were not included. In this study, ARDS resulted from various conditions, with pneumonia being the most common cause (n=20), followed by aspiration (n=10), sepsis (n=4), and other causes (n=6). Peripheral blood and bronchoalveolar lavage (BAL) samples were collected within 48 to 72 hours after intubation. The ARDS group had a higher proportion of males than the HCs. The severity of ARDS was categorized into mild, moderate, and severe subtypes based on the Berlin definition (20). Among a total of 40 patients, 3 (7.5%) had mild disease; 20 (50%) had moderate disease; and 17 (42.5%) had severe disease. Further clinical and laboratory characteristics of the patients are listed in **Table 1**.

**Table 1:** Clinical and laboratory characteristics of ARDS patients.

Characteristic	ARDS		
Total no.	40		
Sex (no. male/no. female)	30/10		
Age (years), mean ± SD	62.8	±	18.6
Clinical variables			
Cause of disease, n (%)			
Pneumonia	20/40 (50)		
Aspiration	10/40 (25)		
Non-pulmonary sepsis	4/40 (10)		
Others	6/40 (15)		
Disease severity, n (%)			
Mild	3/40 (7.5)		
Moderate	20/40 (50)		
Severe	17/40 (42.5)		
Mortality	18/40 (45)		
Mild	0/3 (0)		
Moderate	9/20 (45)		
Severe	9/17 (52.9)		
PEEP (cmH_2_O), mean ± SD	10.4	±	2.94
ECMO	7 (17.5)		
Laboratory variables, mean ± SD			
Leukocyte count (cells/μL)	12,690	±	6,395
Lymphocyte count (cells/μL)	1,227	±	1338.0
Monocyte count (cells/μL)	725.3	±	570.6
Neutrophil count (cells/μL)	10,813	±	6,136
Hemoglobin level (g/dL)	10.30	±	1.78
Platelet count (×10^3^ cells/μL)	180.1	±	110.9
PaCO_2_ (mmHg)	42.9	±	12.1
PaO_2_ (mmHg)	84.3	±	29.9
FiO_2_ (mmHg)	0.69	±	0.19
PaO_2_/FiO_2_ ratio	119	±	52.9
Total protein level (g/dL)	5.40	±	0.98
Albumin (g/dL)	2.57	±	0.98
AST (U/L)	166.3	±	562.9
ALT (U/L)	97.5	±	268.2
BUN (mg/dL)	32.9	±	18.8
Creatinine (mg/dL)	1.43	±	1.58
CRP level (mg/dL)	17.2	±	10.4

ALT, alanine transaminase; ARDS, acute respiratory distress syndrome; AST, aspartate transaminase; BUN, blood urea nitrogen; CRP, C-reactive protein; ECMO, extracorporeal membrane oxygenation; FiO_2_, fraction of inspired oxygen; PaCO_2_, partial pressure of arterial carbon dioxide; PaO_2_, partial pressure of arterial oxygen; PaO_2_/FiO_2_, ratio of the partial pressure of arterial oxygen to the fraction of inspired oxygen; PEEP, positive end expiratory pressure.

### Monoclonal antibodies and flow cytometry

The following mAbs and reagents were used in this study: phycoerythrin (PE)-conjugated anti-CD3, peridinin chlorophyll protein complex (PerCP)-conjugated anti-CD45, allophycocyanin (APC)-conjugated anti-6B11, fluorescein isothiocyanate (FITC)-conjugated anti-Annexin V, FITC-conjugated anti-CD3, PE-conjugated anti-CD69, PE-conjugated anti-programmed cell death-1(PD-1), APC-Cy7-conjugated anti-CD3, PE-conjugated anti-6B11, FITC-conjugated anti-IFN-γ, APC-conjugated anti-IL-4, PerCP-Cy5.5-conjugated anti-IL-17, FITC-conjugated anti-CD14, PE-conjugated anti-CD1d, PE-Cy5-conjugated anti-CD19, APC-conjugated anti-CD3, APC-Cy7-conjugated anti-CD45, FITC-conjugated anti-TNF-α, PE-conjugated anti-IL-1β, FITC-conjugated anti-IL-6, PE-conjugated anti-IL-8, FITC-conjugated mouse IgG isotype, PE-conjugated mouse IgG isotype, and APC-Cy7-conjugated mouse IgG isotype control, PerCP-Cy5.5-conjugated mouse IgG isotype control (all from Becton Dickinson, San Diego, CA). Cells were stained with combinations of appropriate mAbs for 20 minutes at 4°C. Stained cells were analyzed on a Navios flow cytometer using Kaluza software (Beckman Coulter, Brea, CA).

### Isolation of peripheral blood mononuclear cells and identification of NKT cells

Peripheral venous blood samples were collected in heparin-containing tubes. PBMCs were isolated by density-gradient centrifugation using Ficoll-Paque Plus solution (Amersham Bioscience, Uppsala, Sweden). NKT cells were identified phenotypically as CD3^+^6B11^+^ by flow cytometry as previously described (2).

### NKT cell proliferation assay

Proliferative abilities of NKT cells were assayed by flow cytometry as described previously (21). Briefly, freshly isolated PBMCs were suspended in complete media supplemented with 10% fetal bovine serum (FBS; Gibco BRL, Grand Island, NY), seeded into a 24-well plate at density of 1 × 10^6^/well, and then cultured at 37°C in a 5% CO_2_ humidified incubator for 7 days in the presence of IL-2 (100 IU/mL; BD PharMingen, San Jose, CA) and α-GalCer (100 ng/mL; Alexis Biochemicals, Lausen, Switzerland) or 0.1% dimethyl sulfoxide (DMSO) as a control. Cells were harvested and stained with APC-conjugated anti-6B11, PE-conjugated anti-CD3, and PerCP-conjugated anti-CD45 mAbs. Percentages of CD3^+^6B11^+^ NKT cells were determined by flow cytometry using a CD45/SSC gate. Proliferation index was defined as the value of percentage of NKT cells (100 ng/mL α-GalCer) minus percentage of NKT cells (0 ng/mL α-GalCer) on Day 7 divided by percentage of NKT cells on Day 0. It was expressed as fold increase.

To determine the effect of a proinflammatory cytokine cocktail, freshly isolated PBMCs were stimulated with a cytokine cocktail consisting of IL-1β (10 ng/mL; PeproTech, London, UK), IFN-γ (10 ng/mL; PeproTech), IL-6 (50 ng/mL; PeproTech, IL-12 (50 ng/mL; PeproTech), IL-17 (1 ng/mL; PeproTech), IL-18 (50 ng/mL; PeproTech) and TNF-α (5 ng/mL; PeproTech) for 7 days in the presence of IL-2 (100 IU/mL) and α-GalCer (100 ng/mL) or DMSO as control.

### Intracellular cytokine staining

Expression levels of IFN-γ, IL-17 and IL-4 in NKT cells were detected by intracellular cytokine flow cytometry as described previously (22). Briefly, freshly isolated PBMCs (1 × 10^6^/well) were incubated at 37°C in 1 mL complete media consisting of RPMI 1640, 2 mM _L_-glutamine, 100 units/mL of penicillin, and 100 μg/mL of streptomycin supplemented with 10% FBS for 2 hours in the presence of α-GalCer (100 ng/mL) or 0.1% DMSO as control. For intracellular cytokine staining, 1 μL of brefeldin A (GolgiPlug; BD Biosciences, San Diego, CA) for 1 mL of cell culture was added. After incubation at 37°C in a 5% CO_2_ humidified incubator for an additional 4 hours, cells were stained with APC-Cy7-conjugated anti-CD3 and PE-conjugated anti-6B11 mAbs for 20 minutes at 4°C, fixed in 4% paraformaldehyde for 15 minutes at room temperature, and permeabilized with Perm/Wash solution (BD Biosciences) for 10 minutes. Cells were then stained with APC-conjugated anti-IL-4, FITC-conjugated anti-IFN-γ and PerCP-Cy5.5-conjugated anti-IL-17 mAbs for 30 minutes at 4°C and analyzed by flow cytometry.

### Coculture system for human macrophage activity assay

Monocytes were differentiated into macrophage using a coculture system as described previously (23). Briefly, monocytes were isolated from PBMCs at purities of > 95% using CD14 MicroBeads according to the instructions of the manufacturer (Miltenyi Biotec, Bergisch Gladbach, Germany). Monocytes were seeded in a 24-well plate at 5.6 × 10^5^ cells/well and cultured for 6 days in the presence of macrophage colony-stimulating factor (M-CSF; 100 ng/mL; PeproTech). Half medium was replaced at day 3 of culture. NKT cells were isolated from PBMCs using a cell sorter (MoFlo Astrios; Beckman Coulter, Brea, CA). After 6 days, macrophages were cultured with sorted NKT cells (1 × 10^5^ cells/well) in the presence of M-CSF (100 ng/mL), IL-2 (100 IU/mL) and α-GalCer (100 ng/mL) or DMSO as control for 12, 24, and 48 hours. After coculture, 10 μL of brefeldin A (BD Biosciences) was added for intracellular cytokine staining. The final concentration of brefeldin A was 10 μg/mL. After incubation for an additional 4 hours, cells were fixed in 4% paraformaldehyde for 15 minutes at room temperature and permeabilized with Perm/Wash solution (BD Biosciences) for 10 minutes. Cells were then stained with FITC-conjugated anti-TNF-α, FITC-conjugated anti-IL-6, PE-conjugated anti-IL-1β, and PE-conjugated anti-IL-8 mAbs for 30 minutes at 4°C and analyzed by flow cytometry.

### Isolation and culture of alveolar macrophages

Alveolar macrophages were isolated from bronchoalveolar lavage (BAL) of healthy controls as previously described (24, 25). Aspirated BAL fluid was filtered through a cell strainer to remove particulate debris prior to centrifugation. BAL cells were resuspended in RPMI 1640 medium containing 2 mM _L_-glutamine, 100 units/mL of penicillin, and 100 μg/mL of streptomycin supplemented with 10% FBS, and cultured at 1 × 10^6^ cells/mL in a 10 cm dish in the presence of M-CSF (100 ng/mL). After 24 hours, non-adherent cells were removed and adherent cells were cultured with RPMI 1640 medium in the presence of M-CSF for 6 days.

### Coculture system for fibroblast activity assay

A549, human alveolar epithelial cells (ATCC, Manassas, VA) were maintained in RPMI 1640 medium supplemented with 2 mM _L_-glutamine, 100 units/mL of penicillin, and 100 μg/mL of streptomycin and 10% FBS. A transwell system was used to establish the coculture model between AMs and the A549 alveolar epithelial cell line. A549 cells were seeded in a 24-well plate at 5 × 10^4^ cells/well and cultured for 24 hours in the presence or absence of TGF-β (5 ng/mL; Sigma, Saint Louis, MO). NKT cells were isolated from PBMCs using a cell sorter (MoFlo Astrios; Beckman Coulter, Brea, CA). After pretreated with TGF-β, AMs (5 × 10^4^ cells/well) and NKT cells (2 × 10^5^ cells/well) were cocultured in upper chamber for 72 hours in the presence or absence of α-GalCer and conditioned medium (CM) using a 0.4 μm pored transwell. CM was obtained from PBMCs stimulated with α-GalCer for 72 hours.

### Quantitative real-time PCR

Real-time PCR was performed as described previously (26). Briefly, total RNA was extracted from cultured cells using RNAiso Plus Reagent (Takara, Kusatsu, Japan). First-strand cDNA was transcribed from 200 ng of RNA using Superscript RT (Invitrogen) according to the manufacturer’s instructions. To determine the expression levels of specific genes and glyceraldehyde-3-phosphate dehydrogenase (GAPDH, as an internal control), PCRs were performed using TB Green^®^ Premix Ex Taq™ (Takara) in triplicate on a Thermal Cycler Dice TP800 (Takara Bio, Kyoto, Japan) with the following PCR conditions: 15 minutes at 95°C, followed by 40 amplification cycles of 95 °C for 30 seconds, 58 °C for 30 seconds, and 72 °C for 30 seconds. All quantitation was normalized to the levels of endogenous GAPDH. The relative quantification value of each target gene as compared with the calibrator for that target was calculated using 2-(Ct-Cc) (Ct and Cc mean threshold cycle differences after normalizing with respect to GAPDH). Relative expression levels were presented using semi log plots. Sequences of primers used were as follows: for human GAPDH, 5’-GAAGGTGAAGGTCGGAGT-3’ (sense) and 5’-GAAGATGGTGATGGGATTTC-3’ (antisense); for α-SMA, 5’-TCAAATACCCCATTGAACACGG-3’ (sense) and 5’-GGTGCTCTTCAGGTGCTACA-3’ (antisense); for collagen I, 5’-TCTGACTGGAAGAGTGGAGAGTAC-3’ (sense) and 5’-ATCCATCGGTCATGCTCTCG-3’ (antisense); for fibronectin, 5’-CTTTGGTGCAGCACAACTTC-3’ (sense) and 5’-CCTCCTCGAGTCTGAACCAA-3’ (antisense); and for N-cadherin, 5’-CTCCATGTGCCGGATAGC-3’ (sense) and 5’-CGATTTCACCAGAAGCCTCTAC-3’ (antisense).

### Statistics

All comparisons of percentages, absolute numbers, cytokine levels, NKT cell proliferation indices, and expression levels of CD69, PD-1, and Annexin-V were conducted using analysis of covariance (ANCOVA) after adjusting for age and sex, with Bonferroni correction for multiple comparisons. Linear regression analysis was used to explore associations between NKT cell levels and clinical parameters. The Wilcoxon matched-pairs signed-rank test was employed for comparing changes in NKT cell proliferation indices following cytokine stimulation. The Mann-Whitney U test was applied for comparison of CD1d expression between HCs and patients. Paired *t*-tests were utilized for comparing changes in expression levels of proinflammatory cytokines from human macrophages and levels of mesenchymal and fibroblast activation markers from A549. Statistical significance was considered when *P* < 0.05.

### Ethics approval and consent to participate

The study protocol was approved by the Institutional Review Board of Chonnam National University Hospital (IRB No.: CNUH-2021-224). Written informed consent was obtained from all participants in accordance with the Declaration of Helsinki.

## Results

### Reduced numbers of circulating NKT cells in patients with ARDS

**Figure 1:**
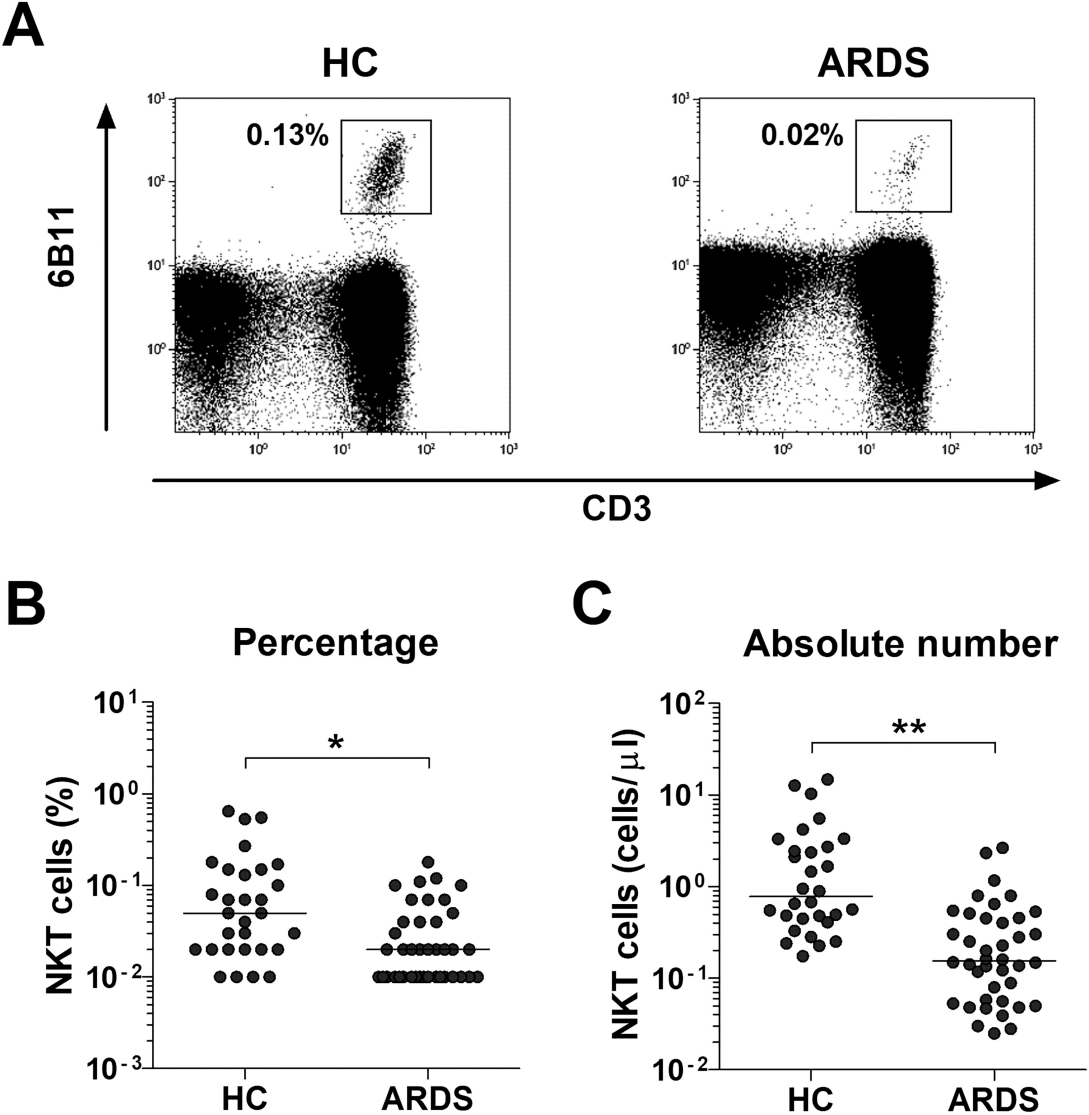
Reduced circulating natural killer T (NKT) cell numbers in the peripheral blood of patients with acute respiratory distress syndrome (ARDS). **(A)** Representative NKT cell percentages as determined by flow cytometry. **(B)** NKT cell percentages among peripheral blood lymphocytes. **(C)** Absolute NKT cell numbers (per microliter of blood). Data for *B* and *C* were obtained from 30 healthy controls (HCs) and 40 patients with ARDS. Symbols represent individual subjects, and horizontal lines are median values. *P < 0.005, **P < 0.0005 by analysis of covariance (ANCOVA).

Percentages and absolute numbers of circulating NKT cells were determined in 40 ARDS patients and 30 HCs using flow cytometry (**Figure 1A**). The absolute numbers of circulating NKT cells were calculated by multiplying each cell fraction by the total number of lymphocytes (per microliter of peripheral blood). Patients with ARDS exhibited significantly lower percentages (median 0.02% vs. 0.05%, *P* < 0.005) and absolute numbers (median 0.16 cells/μL vs. 0.79 cells/μL, *P* < 0.0005) of NKT cells compared to HCs (**Figures 1B, C**).

### Relationship between circulating NKT cell levels and clinical parameters in patients with ARDS

Regression analysis was performed to assess the clinical significance of NKT cells in ARDS patients. This analysis identified significant correlations between log-transformed absolute NKT cell counts and various clinical parameters, including lymphocyte count, monocyte count, PaO_2_/FiO_2_ ratio, albumin, and C-reactive protein levels (*P* = 0.0001, *P* = 0.035, *P* = 0.012, *P* = 0.044, and *P* = 0.03, respectively). However, there were no statistically significant differences in disease severity and mortality among ARDS patients (**Table 2**).

**Table 2:** Regression coefficients for log-transformed absolute NKT cell numbers with respect to clinical and laboratory findings in ARDS patients.

Variables	β	SE	*P* value
Sex (male)	0.0001	0.192	0.998
Age (years)	-0.003	0.005	0.471
Disease severity	-0.168	0.133	0.213
Mortality	-0.21	0.164	0.209
PEEP (cmH_2_O)	-0.054	0.029	0.071
ECMO	-0.412	0.209	0.056
Leukocyte count (cells/μL)	0.0001	0.0001	0.595
Lymphocyte count (cells/μL)	0.001	0.0001	0.0001
Monocyte count (cells/μL)	0.0001	0.0001	0.035
Neutrophil count (cells/μL)	-0.0001	0.0001	0.836
Hemoglobin (g/dL)	0.051	0.047	0.283
Platelet count (×10^3^ cells/μL)	0.001	0.001	0.425
PaCO_2_ (mmHg)	0.001	0.007	0.912
PaO_2_ (mmHg)	-0.004	0.003	0.149
FiO_2_ (mmHg)	-0.532	0.424	0.217
PaO_2_/FiO_2_ ratio	0.004	0.001	0.012
Total protein (g/dL)	0.163	0.082	0.054
Albumin (g/dL)	0.381	0.183	0.044
AST (U/L)	0.0001	0.0001	0.095
ALT (U/L)	0.0001	0.0001	0.217
BUN (mg/dL)	-0.002	0.004	0.612
Creatinine (mg/dL)	-0.011	0.054	0.838
CRP (mg/dL)	-0.017	0.008	0.030

ARDS, Acute respiratory distress syndrome; ALT, alanine transaminase; AST, aspartate transaminase; β, regression coefficient; BUN, blood urea nitrogen; CI, confidence intervals; CRP, C-reactive protein; ECMO, extracorporeal membrane oxygenation; FiO_2_, fraction of inspired oxygen; NKT, natural killer T; PaCO_2_, partial pressure of arterial carbon dioxide; PaO_2_, partial pressure of arterial oxygen; PaO_2_/FiO_2_, ratio of the partial pressure of arterial oxygen to the fraction of inspired oxygen; PEEP, positive end expiratory pressure; SE, standard error.

### Activation and alteration of cytokine-producing profiles of NKT cells in patients with ARDS

**Figure 2:**
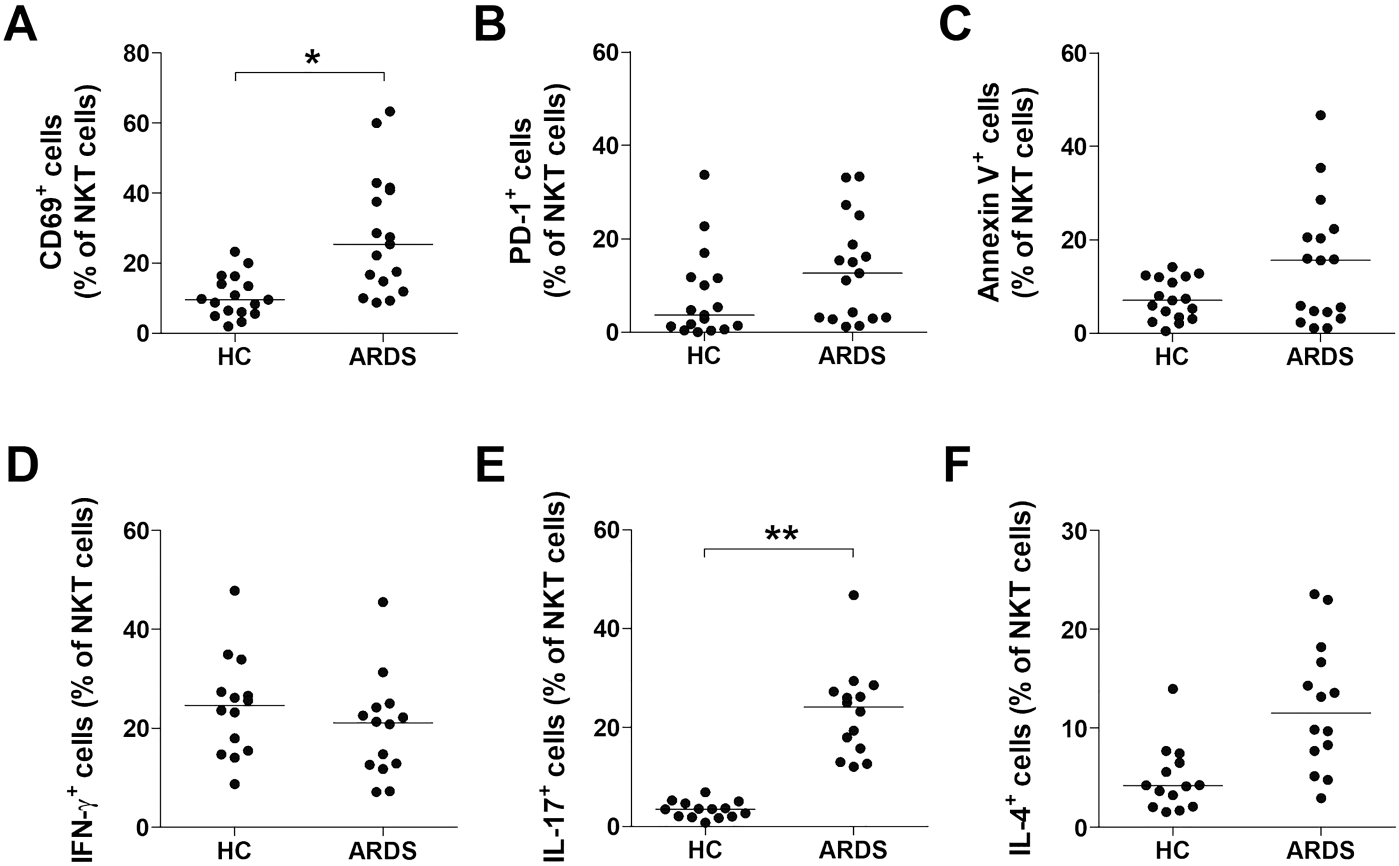
Activation of natural killer T (NKT) cells in acute respiratory distress syndrome (ARDS) patients. Data in panels **(A–C)** were obtained from 17 healthy controls (HCs) and 17 patients with ARDS. **(D–F)** Production of IFN-γ, IL-17 and IL-4 in the NKT cell population. Freshly isolated peripheral blood mononuclear cells (1×10^6^/well) were cultured for 2 hours in the presence in the of α-galactosylceramide (100 ng/mL) or 0.1% dimethyl sulfoxide as control. Percentages of IFN-γ, IL-17 and IL-4 were calculated among total NKT cell number using an NKT cell. Data in **(D–F)** were obtained from 14 HCs and 14 ARDS patients. Symbols represent individual subjects. In **(A–F)**, horizontal bars show the median. *P < 0.05, **P < 0.005 by analysis of covariance (ANCOVA).

To investigate whether the deficiency of circulating NKT cells was associated to activation-induced cell death, we assessed the expression levels of CD69, PD-1, and annexin-V in circulating NKT cells from 17 ARDS patients and 17 HCs using flow cytometry (**Figures 2A–C**). The percentage of CD69^+^ NKT cells was significantly elevated in ARDS patients compared to HCs (median 25.4% vs. 9.55%, *P* < 0.05; **Figure 2A**), whereas the percentages of PD-1^+^ and annexin-V^+^ NKT cells were similar (**Figures 2B, C**). Furthermore, we analyzed the cytokine release profile of NKT cells. PBMCs from 14 ARDS patients and 14 HCs were incubated with α-GalCer or with 0.1% DMSO as a control for 2 hours. Intracellular flow cytometry (**Figures 2D–F**) demonstrated a significantly higher percentage of IL-17-producing NKT cells in ARDS patients in comparison to HCs (median 24.1% vs. 3.50%, *P* < 0.005; **Figure 2E**), while the levels of IFN-γ^+^ and IL-4^+^ NKT cells did not differ significantly between the groups (**Figures 2D, F**).

### Impaired proliferative response of NKT cells to α-GalCer in patients with ARDS

**Figure 3:**
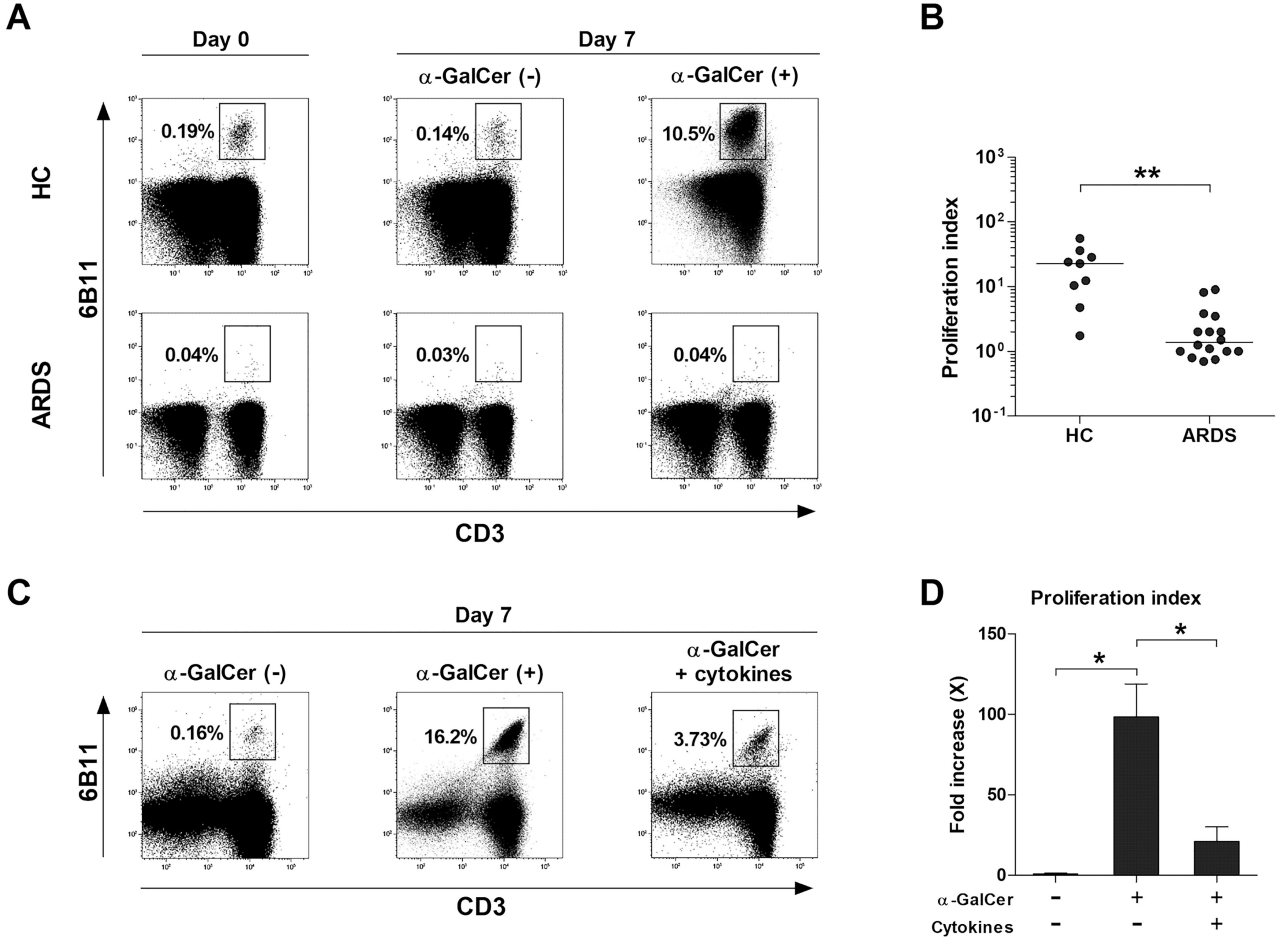
Impaired proliferative response of natural killer T (NKT) cells to α-GalCer in acute respiratory distress syndrome (ARDS) patients. **(A, B)** Freshly isolated peripheral blood mononuclear cells (PBMCs; 1 × 10^6^/well) from 12 HCs and 16 ARDS patients were cultured for 7 days in the presence of IL-2 (100 IU/ml) and α-GalCer (100 ng/mL) or 0.1% dimethyl sulfoxide. **(A)** Representative NKT cell percentages determined by flow cytometry. **(B)** Proliferation indices of NKT cells. Symbols represent individual subjects. Horizontal bars show the median **P < 0.005 by the ANCOVA test. **(C, D)** Effects of stimulation with proinflammatory cytokine cocktail on the proliferative responses of NKT cells to α-GalCer were determined. Data in **(C, D)** were obtained from 6 HCs. Values are presented as mean ± SEM. *P < 0.05 by Wilcoxon matched-pairs signed-rank test.

To examine the proliferative response of NKT cells to α-GalCer in patients with ARDS, PBMCs from 16 ARDS patients and 12 HCs were cultured with IL-2 and α-GalCer or 0.1% DMSO for seven days. The percentages of NKT cells and their proliferation indices were assessed using flow cytometry. Upon stimulation with α-GalCer, a significant increase in the percentage of NKT cells was observed in HCs, from 0.19% on day 0 to 10.5% on day 7. However, in ARDS patients, the NKT cell percentage remained constant at 0.04% on both day 0 and day 7, despite α-GalCer stimulation (**Figure 3A**). The overall proliferation indices were significantly lower in ARDS patients compared to HCs (median 1.38 vs. 23.3, *P* < 0.005; **Figure 3B**). Given the elevated levels of inflammatory cytokines in ARDS (27, 28), the effect of these cytokines on NKT cell proliferation in response to α-GalCer was examined. NKT cells from six HCs were divided into three groups: unstimulated, stimulated with α-GalCer alone, and stimulated with both α-GalCer and proinflammatory cytokines. After seven days, the percentages of NKT cells in these groups were 0.16%, 16.2%, and 3.73%, respectively (**Figure 3C**). The proliferation index was significantly higher in the group stimulated with α-GalCer alone compared to the unstimulated group (median 85.9 vs. 0.72, *P* < 0.05). However, stimulation with both α-GalCer and proinflammatory cytokines significantly reduced the proliferation index compared to α-GalCer stimulation alone (median 11.2 vs. 85.9, *P* < 0.05; **Figure 3D**). These findings indicate that the proliferative capacity of NKT cells in response to α-GalCer is partially impaired by proinflammatory cytokines.

### The mechanism involved in the poor response to α-GalCer in ARDS patients

**Figure 4:**
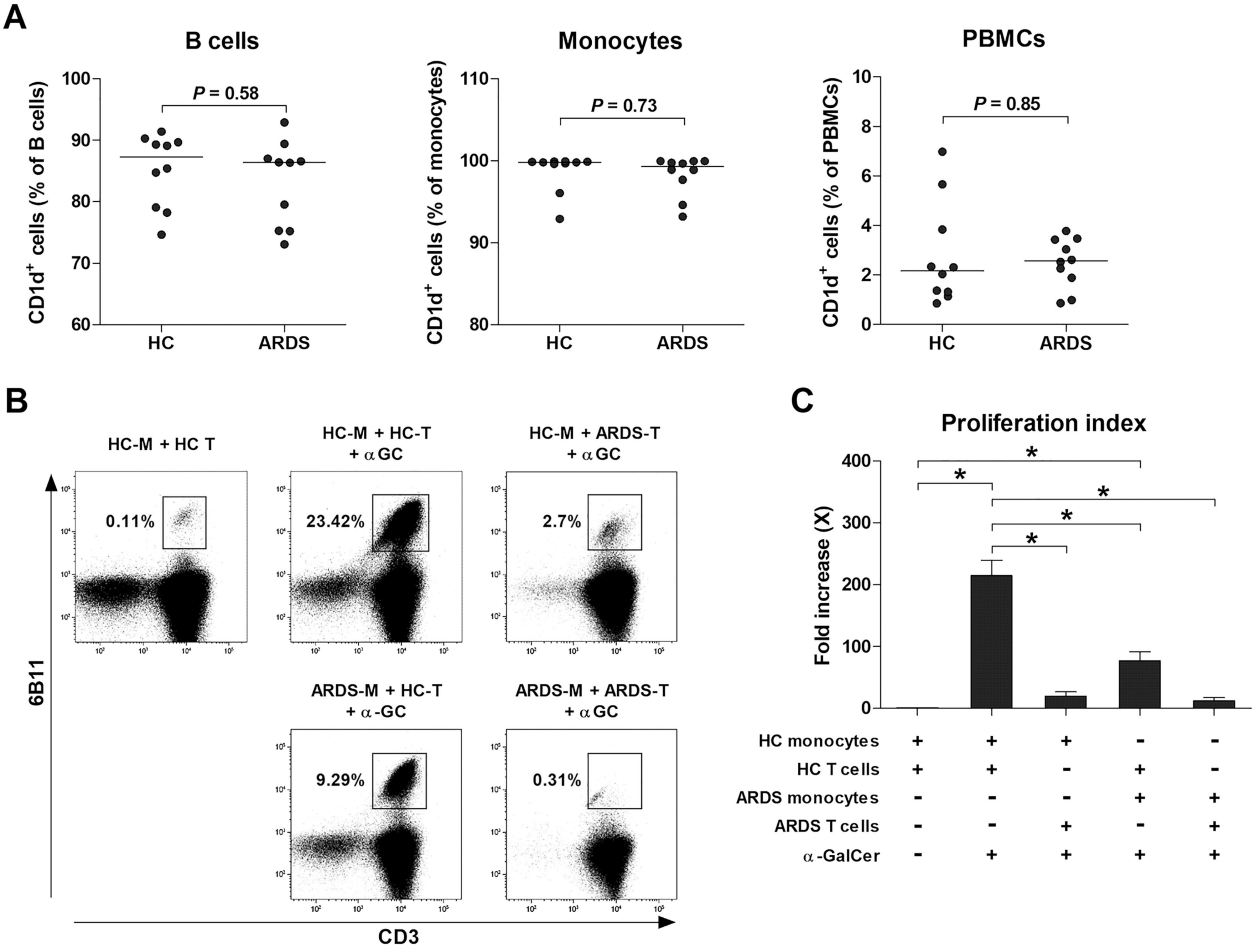
The mechanism involved in poor response to α-GalCer in acute respiratory distress syndrome (ARDS) patients. **(A)** CD1d expression by peripheral blood mononuclear cells (PBMCs) from 10 HCs and 10 ARDS patients. Symbols represent individual subjects. Horizontal bars show the median. *P* values by Mann-Whitney U test. **(B, C)** Abnormalities of the NKT cells and monocytes of ARDS patients. APCs and T cells were cross co-cultured with α-GalCer. Monocytes were used as a source of APCs. Freshly isolated monocytes (M; 1 × 10^5^ cells/well) were co-cultured with T cells (T; 9 × 10^5^ cells/well) for 7 days as described in Methods section. **(B)** Representative NKT cell percentages determined by flow cytometry. **(C)** Proliferation indices of NKT cells. Data in **(C)** were obtained from 3 HCs and 3 ARDS patients. Values are presented as mean ± SEM. *P < 0.05 by paired *t*-test.

Impaired NKT cell function in ARDS might result from abnormalities in the recognition of α-GalCer on CD1d or in the interaction with immune cells. To assess the impact of CD1d-expressing cells, PBMCs from 10 HCs and 10 ARDS patients were examined, showing no significant differences in the expression of CD1d on monocytes, B cells, or PBMCs (**Figure 4A**). Subsequent analysis concentrated on the effect of ARDS-derived immune cells on NKT cell activation. The inclusion of monocytes and T cells from HCs served as the control. Cross-culturing with α-GalCer for seven days resulted in a notable increase in activated NKT cells when α-GalCer was introduced to the control (median 1.0 vs. 200.9, *P* < 0.05). Nevertheless, a significant reduction was observed when monocytes or T cells from ARDS patients substituted the control, with the minimal NKT cell percentage noted when both were replaced (**Figures 4B, C**). These observations suggest that diminished NKT cell activity in ARDS could stem from defective interactions with immune cells and proinflammatory cytokines.

### Increased and activated NKT cells in BAL fluid from patients with ARDS promote the expression of proinflammatory cytokines in human macrophages

**Figure 5:**
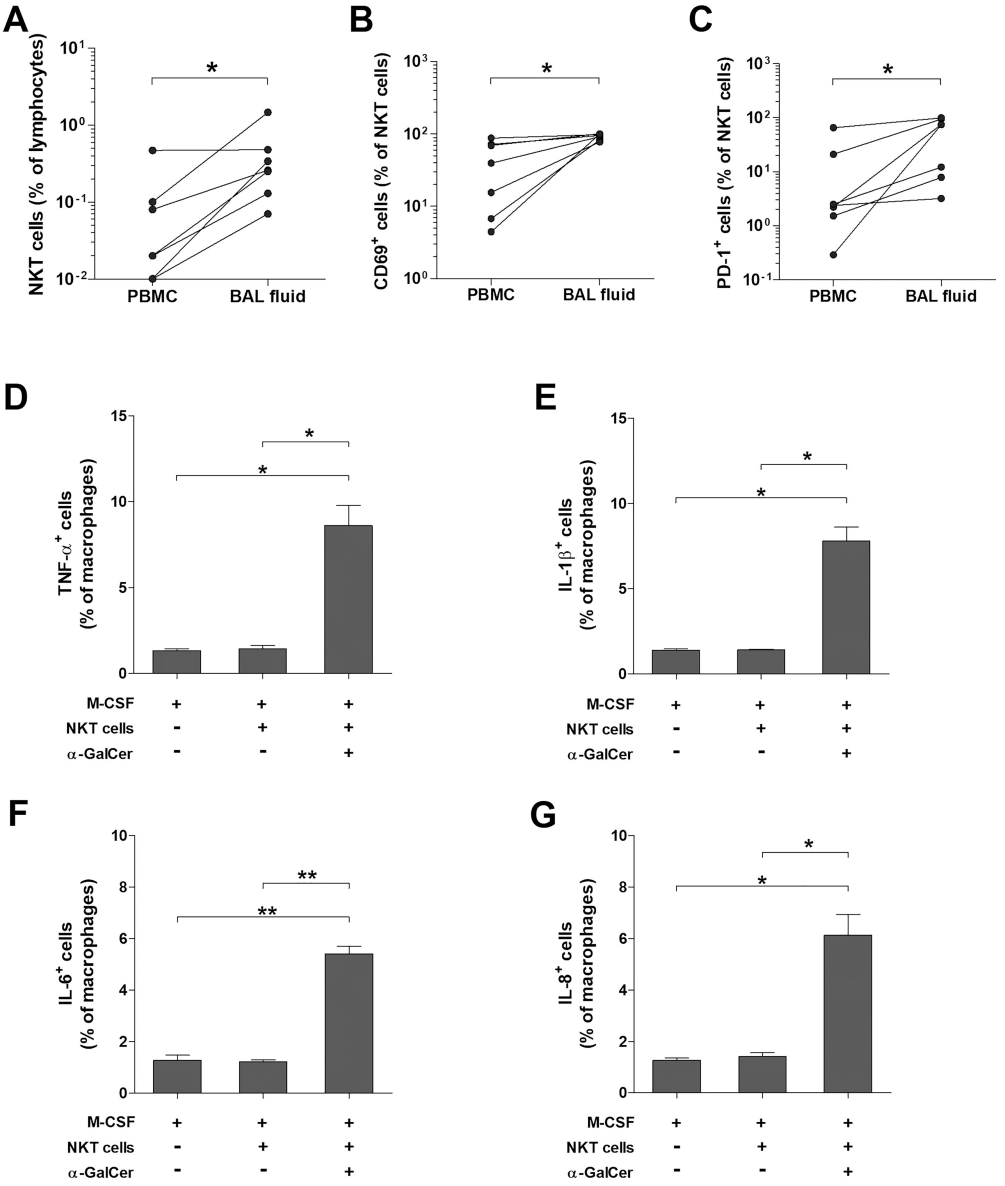
Expression of proinflammatory cytokines in human macrophage by activated NKT cells. Paired samples of peripheral blood and BAL fluid were obtained from ARDS patients, and then the percentages of total NKT cells **(A)**, CD69^+^ NKT cells **(B)**, and PD-1^+^ NKT cells **(C)** were determined by flow cytometry. Data in were obtained from 6 patients with ARDS, respectively. Symbols represent individual patients. *P < 0.05 by Wilcoxon matched-pairs signed-rank test. Purified monocytes (2.5 × 10^6^ cells/well) were cultured for 7 days in the presence of M-CSF (100 ng/mL. Macrophages were cultured with sorted NKT cells (1 × 10^6^ cells/well) in the presence of M-CSF, IL-2 and α-GalCer, or DMSO as a control for 24 hours. **(D–G)** Expression of TNF-α, IL-1β, IL-6 and IL-8 in macrophage determined by intracellular flow cytometry. Data in **(D–G)** were from 3 independent experiments. Values are expressed as the mean ± SEM. *P < 0.005, **P < 0.0005 by unpaired *t*-test.

To investigate the association between the reduced numbers of circulating NKT cells in patients with ARDS and their accumulation in BALF, we collected paired samples of peripheral blood and BALF from seven patients. The percentages of NKT cells were significantly higher in BALF than in peripheral blood (median 0.26 vs. 0.02, *P* < 0.05; **Figure 5A**). Furthermore, the percentages of CD69 and PD-1 expressing NKT cells in BALF were higher compared with peripheral blood (median 95.2 vs. 39.5, *P* < 0.05, and 74.7 vs. 2.38, *P* < 0.05, respectively; **Figures 5B, C**).

ARDS patients are known to undergo three phases: the exudative, proliferative, and fibrotic phases (26). Considering the diverse immunological functions of NKT cells, we posited they might play different roles in each phase of ARDS. To determine whether activated NKT cells could induce macrophages, crucial in the exudative phase, to produce proinflammatory cytokines, we cultured purified monocytes with M-CSF for seven days to differentiate them into macrophages. These macrophages were activated under three conditions: M-CSF alone, M-CSF with resting NKT cells, and M-CSF with α-GalCer-activated NKT cells. The percentages of macrophages expressing TNF-α, IL-1β, IL-6, and IL-8 were significantly elevated in the presence of M-CSF with α-GalCer-activated NKT cells compared to M-CSF alone (median 8.85 vs. 1.33, *P* < 0.005, for TNF-α; 7.19 vs. 1.41, *P* < 0.005, for IL-1β; 5.18 vs. 1.25, *P* < 0.0005, for IL-6; and 5.8 vs. 1.29, *P* < 0.0005, for IL-8, respectively) or M-CSF with resting NKT cells (median 8.85 vs. 1.35, *P* < 0.005, for TNF-α; 7.19 vs. 1.42, *P* < 0.005, for IL-1β; 5.18 vs. 1.21, *P* < 0.0005, for IL-6; and 5.8 vs. 1.34, *P* < 0.005, for IL-8, respectively; **Figures 5D–G**).

### Interaction between alveolar macrophages and NKT cells may have the potential to stimulate lung fibroblasts

**Figure 6:**
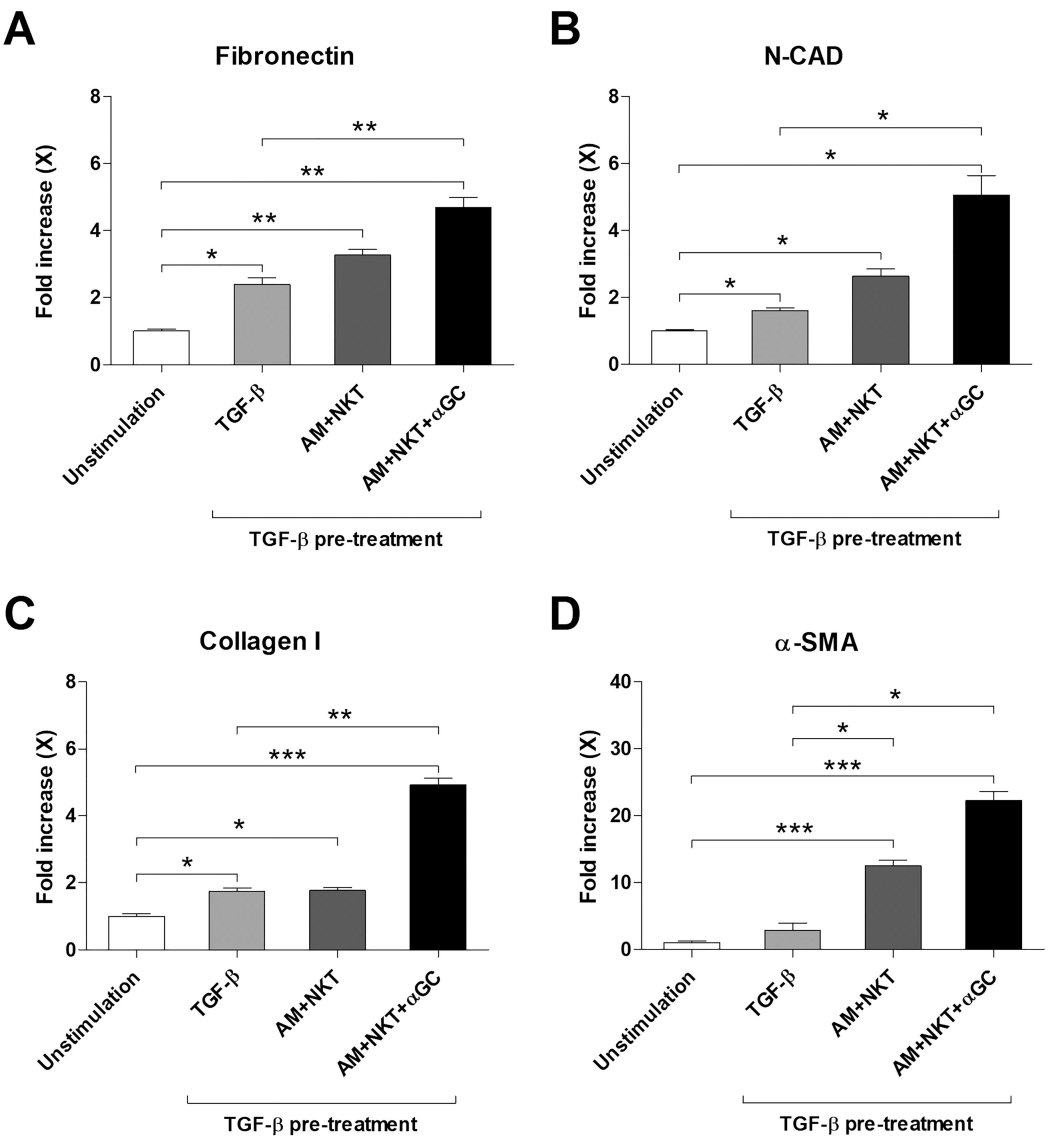
Increased expression of mesenchymal markers and fibroblast activation markers following coculture with NKT cells of A549 cells. A549 cells were pretreated with or without TGF-β (5 ng/mL) and then coculture with alveolar macrophages (5 × 10^4^ cells/well) and NKT cells (2 × 10^5^ cells/well) for 72 hours in the presence or absence of α-GalCer using transwell. Expression of fibronectin **(A)**, N-cadherin **(B)**, collagen I **(C)** and α-smooth muscle actin (SMA) **(D)** in A549 was measured by real-time PCR. The changes of expression level are expressed as fold increase compared to unstimulated controls. Data were from 3 independent experiments. Values are expressed as the mean ± SEM. *P < 0.05, **P < 0.01, ***P < 0.005 by paired *t*-test.

To examine the effect of alveolar macrophages and NKT cells on fibroblasts, which play a crucial role in the proliferative and fibrotic phases, the A549 human alveolar epithelial cell line was pretreated with TGF-β for 24 hours to induce epithelial-mesenchymal transition (EMT) (30, 31). Following this, they were co-cultured with NKT cells and alveolar macrophages from BALF of HCs for 72 hours, in the presence or absence of α-GalCer in the transwell system. Subsequently, the expression levels of fibronectin, N-cadherin (N-CAD), collagen I, and α-smooth muscle actin (SMA) were quantified using real-time PCR. The results revealed significant increases in the expression of fibronectin, N-CAD, and collagen I in the TGF-β-induced EMT A549 cell line compared to the unstimulated group (median 2.33 vs. 0.97, *P* < 0.05, for fibronectin; 1.64 vs. 1.01, *P* < 0.05, for N-CAD; 1.7 vs. 0.97, *P <* 0.05, for collagen I). Additionally, the addition of alveolar macrophages and NKT cells with α-GalCer to the EMT environment resulted in notable increases in the expression of fibronectin, N-CAD, collagen I, and α-SMA compared to controls without these cells (median 4.64 vs. 2.33, *P* < 0.01, for fibronectin; 4.56 vs. 1.64, *P* < 0.05, for N-CAD; 5.09 vs. 1.7, *P* < 0.005, for collagen I; 21.7 vs. 2.37, *P* < 0.05, for α-SMA, respectively; **Figures 6A–D**).

## Discussion

To our knowledge, this study represents the first to explore the levels and functions of human NKT cells in ARDS, evaluate their clinical significance, and examine their relationship with macrophages and fibroblasts involved in ARDS pathogenesis. We discovered a deficiency of circulating NKT cells in patients with ARDS, accompanied by an increased expression of CD69 and enhanced IL-17 production. The response of NKT cells to α-GalCer was diminished in ARDS patients. Furthermore, co-culturing NKT cells with monocytes or T cells from ARDS patients led to a diminished response to α-GalCer. This reduced response was not observed when tested against CD1d-expressing B cells and monocytes. We also observed that activated NKT cells were more prevalent in BALF than PBMC in ARDS patients. With α-GalCer stimulation, the co-presence of NKT cells with macrophages in M-CSF significantly increased the expression of inflammatory cytokines compared to M-CSF alone. Moreover, a notable increase in several ECM expressions was observed when alveolar macrophages and NKT cells with α-GalCer were introduced into the EMT condition. Together, these results highlight the potential role of NKT cells as a vital immune modulator in the progression of lung inflammation, the healing of damaged tissue, and fibrosis in ARDS patients.

Our data indicate that ARDS patients exhibit a reduction in circulating NKT cells. Several studies have shown a reduction in NKT cell numbers in various infectious and systemic inflammatory diseases (21, 32, 33). Of note, recent research on COVID-19 patients revealed decreased NKT cell frequencies in both the acute and recovery phases, with the reduction during the acute phase positively correlating with the PaO_2_/FiO_2_ ratio (32, 34). These outcomes align with ours, suggesting that the decreased peripheral NKT cell numbers might reflect deteriorating lung function in ARDS patients.

CD69 acts as an early activation marker, whereas PD-1 and Annexin-V are linked to anergy/exhaustion or late activation (35–37). Our research shows an increase in CD69 expression in circulating NKT cells of ARDS patients, aligning with findings in trauma patients (38). Similarly, MAIT cells, another unconventional T cell subset, display analogous activation patterns in both ARDS and COVID-19 patients (39, 40). However, unlike MAIT cells, our analysis of PD-1 and Annexin-V in peripheral NKT cells from ARDS patients indicated an increasing trend without achieving statistical significance. This observation might be due to the relatively earlier activation of NKT cells in ARDS patients compared to MAIT cells, necessitating further investigation. Our results also demonstrated elevated IL-17 secretion in circulating NKT cells from ARDS patients. Various studies have indicated the induction of IL-17-producing NKT cells in a proinflammatory environment prior to their migration into peripheral tissues (41). These findings could support our data, suggesting that the inflammatory state in ARDS prompts NKT cells to secrete IL-17. Collectively, NKT cells in ARDS patients are subject to early activation, secreting significant amount of IL-17, which may intensify the inflammatory environment in ARDS.

Our results indicate that NKT cells in ARDS patients exhibited an impaired proliferative response to α-GalCer. This hyporesponsiveness was replicated by treating NKT cells from HCs with a cytokine cocktail that mimics the ARDS condition. Similar reductions in activity against α-GalCer have been observed in other inflammatory and infectious diseases (21, 38). The currently known modes of NKT cell activation involve antigen presentation via CD1d or cytokine induction (42). In exploring the impact of cytokines on NKT cell activation, we treated NKT cells derived from healthy controls with proinflammatory cytokines implicated in the pathogenesis of ARDS (43), revealing a significant reduction in the α-GalCer response. These findings lead us to speculate that the cytokine storm environment in ARDS may contribute to inhibiting the proliferative function of NKT cells.

Our data showed no significant difference in the number of B cells and monocytes expressing CD1d in patients with ARDS, indicating that the level of APCs or antigen presentation via CD1d itself may not be a decisive factor in the diminished NKT cell response in patients with ARDS. Interestingly, the response to α-GalCer significantly decreased when monocytes or T cells from ARDS patients were treated, respectively, compared to control. Previous studies have demonstrated that when NKT cells were stimulated with cytokines alone without TCR stimulation, NKT cells predominantly secreted IFN-r rather than IL-4 (42). In our study, however, NKT cells from ARDS patients tended to secrete IL-4. Collectively, the diminished NKT cell response in ARDS may result from aberrant interactions with immune cells and the influence of proinflammatory cytokines on TCR recognition rather than CD1d expression alone.

This study demonstrated an increased frequency of NKT cells with elevated expression of both CD69 and PD-1 in the BALF compared with peripheral blood in ARDS patients. A similar pattern was observed in MAIT cells of ARDS patients (39). The source of these enhanced NKT cells in BALF is uncertain, whether due to the expansion of tissue-resident NKT cells or the recruitment of circulating NKT cells. However, current evidence indicates that NKT cells can migrate from blood to tissues under different conditions (42). Notably, the BAL from patients with ARDS or COVID-19 exhibited elevated levels of CCL2/MCP-1 and CCL20/MIP-3α (44–46), and research in the context of cancer has demonstrated the migration of NKT cells via these chemokines (47, 48). This information suggests a plausible mechanism by which circulating NKT cells might migrate into the lungs of ARDS patients to engage in various immunological interactions.

The three phases of ARDS, namely the exudative, proliferative, and fibrotic phases, are well recognized (3, 6). Macrophages, characterized by their M1 and M2 phenotypes, serve as effector cells in all phases of ARDS (4, 49). Our study revealed that α-GalCer-activated NKT cells induced monocyte-derived macrophages to secrete TNF-α, IL-1β, IL-6, and IL-8, cytokines typically associated with M1 macrophages (50). These findings, together with our investigations into the role of MAIT cells in ARDS (39), indicate that activated NKT and MAIT cells might contribute to the M1 polarization of macrophages, thus aggravating inflammation during the exudative phase.

Furthermore, this study showed that treatment of A549 cells with TGF-β induced the EMT state, and co-culturing alveolar macrophages and NKT cells heightened the expression of mesenchymal markers and ECM involved in the proliferative and fibrotic phases. This effect was intensified by the addition of α-GalCer. EMT is triggered when epithelial cells are damaged and the inflammatory response remains unresolved (53). TGF-β plays a pivotal role in this process, with its elevated levels observed in BALF in cases of ARDS and COVID-19 (54, 55). Activated by TGF-β, myofibroblasts are essential in remodeling damaged tissue (9, 56). Concurrently, the deposition of fibronectin, collagen I, and α-SMA facilitates lung tissue healing during the proliferative phase; however, excessive accumulation might result in lung tissue fibrosis (5, 29, 51, 52). While our findings suggest that TGF-β-activated myofibroblasts interact with alveolar macrophages and NKT cells, leading to ECM accumulation, the direct functional roles of NKT cells on macrophages and fibroblasts remain to be fully elucidated, which necessitates further study. In summary, our results indicate interactions among NKT cells, macrophages, and fibroblasts, and the significance of their modulation in controlling ARDS. However, further investigations are needed to elucidate the specific actions of NKT cells on these cells.

Recent studies have demonstrated the clinical potential of NKT cell therapy. For instance, two groups engineered allogenic iNKT cells and administered them to patients with COVID-19, revealing anti-inflammatory effects and favorable clinical outcomes (57, 58). Considering our findings that NKT cells are numerically and functionally abnormal in ARDS, administering sufficient number of engineered NKT cells with anti-inflammatory properties could potentially influence the overall immune response and improve clinical outcomes in ARDS patients. Further studies across various etiologies, including non-COVID-19 cases, are necessary to validate these findings. Nevertheless, NKT cell-based therapy shows promise as a viable treatment option for ARDS.

The study is limited by several factors. Firstly, the number of paired samples (BALF and peripheral blood) was small, which restricts the generalizability of the findings. Secondly, the diverse etiologies of ARDS might impact the behavior of NKT cells, potentially confounding the results. Thirdly, changes in immune status throughout the disease course were not identified. Future investigations are warranted to investigate these aspects thoroughly.

In conclusion, this study demonstrated that circulating NKT cells are numerically deficient but functionally active in patients with ARDS. These NKT cells exhibit a diminished response to α-GalCer because of increased proinflammatory cytokines and abnormal interactions with other immune cells in the ARDS state. Additionally, the increased number of NKT cells in the BALF of ARDS patients promotes the production of proinflammatory cytokines by macrophages and also induces the expression of ECM proteins by fibroblasts in collaboration with macrophages. Our novel findings suggest that NKT cells may play a role in the progression of ARDS, underscoring their importance as a potential therapeutic target for ARDS management.

## Data Availability

The raw data supporting the conclusions of this article will be made available by the authors, without undue reservation.
