# Supplementary material for: Deficiency and dysfunctional roles of natural killer T cells in patients with ARDS
Source: Front Immunol. 2024 Aug 30;15:1433028. doi: 10.3389/fimmu.2024.1433028 (PMC11392733; doi:10.3389/fimmu.2024.1433028)
Supplement: Supplementary file 1 [file DataSheet1.pdf]

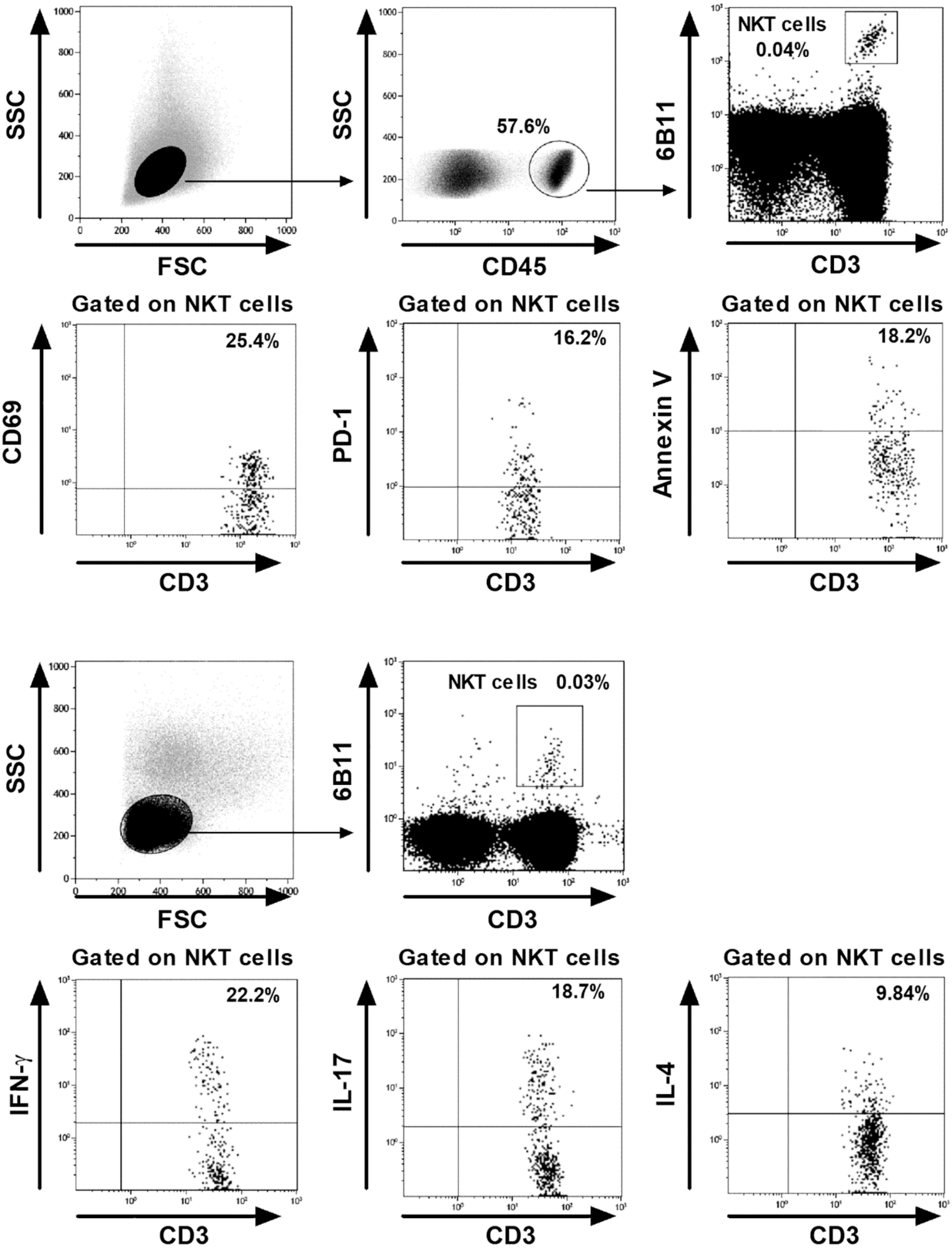

Supplementary Figure 1. Gating strategy for flow cytometry analysis. (A) Representative dot plots for expression of CD69, PD-1, and annexin V in NKT cells. Freshly isolated PBMCs from ARDS patients were stained with APC-conjugated anti-6B11, FITC-conjugated annexin V, FITC-conjugated anti-CD3, PerCP-conjugated anti-CD45, PE-conjugated anti-CD3, PE-conjugated anti-CD69, and PE-conjugated anti-PD-1 mAbs and then analyzed by flow

cytometry. (B) Representative dot plots for production of interferon (IFN)-γ, interleukin (IL)-17, and IL-4 in NKT cells. Freshly isolated peripheral blood mononuclear cells (PBMCs; 1 × 10^6/well) from ARDS patients were incubated for 2 hr in the presence of α-GalCer. Cells were stained with APC-Cy7-conjugated anti-CD3, APC-conjugated anti-IL-4, FITC-conjugated anti-IFN-γ, PE-conjugated anti-6B11, and PerCP-Cy5.5-conjugated anti-IL-17 mAbs and then analyzed by flow cytometry.
